# Ring attractor bio-inspired neural network for social robot navigation

**DOI:** 10.3389/fnbot.2023.1211570

**Published:** 2023-08-31

**Authors:** Jesús D. Rivero-Ortega, Juan S. Mosquera-Maturana, Josh Pardo-Cabrera, Julián Hurtado-López, Juan D. Hernández, Victor Romero-Cano, David F. Ramírez-Moreno

**Affiliations:** ^1^Department of Engineering, Universidad Autónoma de Occidente, Cali, Colombia; ^2^Department of Mathematics, Universidad Autónoma de Occidente, Cali, Colombia; ^3^School of Computer Science and Informatics, Cardiff University, Cardiff, United Kingdom; ^4^Robotics and Autonomous Systems Laboratory, Faculty of Engineering, Universidad Autonoma de Occidente, Cali, Colombia; ^5^Rimac Technology, Zagreb, Croatia; ^6^Department of Physics, Universidad Autónoma de Occidente, Cali, Colombia

**Keywords:** bio-inspired navigation, robot guidance, obstacle avoidance, decision-making, motor control, ring attractor networks, social navigation

## Abstract

**Introduction:**

We introduce a bio-inspired navigation system for a robot to guide a social agent to a target location while avoiding static and dynamic obstacles. Robot navigation can be accomplished through a model of ring attractor neural networks. This connectivity pattern between neurons enables the generation of stable activity patterns that can represent continuous variables such as heading direction or position. The integration of sensory representation, decision-making, and motor control through ring attractor networks offers a biologically-inspired approach to navigation in complex environments.

**Methods:**

The navigation system is divided into perception, planning, and control stages. Our approach is compared to the widely-used Social Force Model and Rapidly Exploring Random Tree Star methods using the Social Individual Index and Relative Motion Index as metrics in simulated experiments. We created a virtual scenario of a pedestrian area with various obstacles and dynamic agents.

**Results:**

The results obtained in our experiments demonstrate the effectiveness of this architecture in guiding a social agent while avoiding obstacles, and the metrics used for evaluating the system indicate that our proposal outperforms the widely used Social Force Model.

**Discussion:**

Our approach points to improving safety and comfort specifically for human-robot interactions. By integrating the Social Individual Index and Relative Motion Index, this approach considers both social comfort and collision avoidance features, resulting in better human-robot interactions in a crowded environment.

## 1. Introduction

In recent years, robots have become increasingly important in diverse applications, ranging from search and rescue operations to medical assistance (Murphy et al., 2008; Pavithra and Yadav, 2022). To enable autonomous operation, two of the most fundamental and essential tasks of robots are navigation and sensing (Cook and Zhang, 2020). Sensing involves the robot collecting data such as position, temperature, range, and pressure in order to make informed decisions while autonomous navigation involves the robot being able to determine its own route based on the environment.

Robots navigation can be accomplished through a model of ring attractor networks. These ring-shaped networks are a powerful tool for modeling cognitive functions that involve circular variables. Ring attractors refer to neural architectures that encode spatial information in a circular or ring-like structure. They are composed of a group of neurons arranged in a closed loop, where each neuron represents a particular location or direction (Kakaria and de Bivort, 2017). The connectivity pattern between these neurons has a ring-like topology and enables the generation of stable activity patterns that can represent continuous variables such as heading direction or position (Skaggs et al., 1995; Knierim and Zhang, 2012; Angelaki and Laurens, 2020). The network dynamics are such that the activity of the neurons on the ring forms a bump, which can move around the ring. The position of the bump on the ring represents the value of the encoded circular variable.

In developing effective navigation systems for robots, ring attractor models have been employed to enable effective path planning, obstacle avoidance, and goal-directed behavior (Chen and Mo, 2019; Robinson et al., 2022; Yuan et al., 2022). Chen and Mo (2019) employed a ring attractor model as a key component of their robotic navigation system. The model encodes and maintains the heading direction of the robot and also enabled the robot to navigate its environment while keeping a stable orientation and exhibiting goal-oriented behaviors. By leveraging the circular structure of the ring attractor, the robot could represent continuous angular variables, such as heading direction, in a robust and efficient manner. Robinson et al. (2022) explored the use of a ring attractor network in the context of orientation estimation during translation in insects. Their research serves as a significant proof-of-concept for a robotics navigation algorithm with a focus on achieving a low size, weight, and power (SWaP) profile. By incorporating these attractor networks they successfully demonstrated the feasibility and potential of employing orientation estimation in a ring attractor network to enable efficient and lightweight navigation algorithms for robotic systems.

There are relevant approaches that do not take into account the neural ring attractors dynamics and are built on considering neurons not like point units but as structured units, compartment-ensembled, formed at least by dendritic trees and soma, each one with its own complex spatiotemporal dynamics, that can provide advantages on sensory processing and spatial navigation for robots with neuromorphic design. Yang et al. (2023a) developed a learning algorithm based on hybrid dendritic mechanisms for spiking-neural networks with multi-compartmental units that lead to higher learning performances under certain structural constraints, reduced synaptic operations, and reduced power consumption on work. Yang et al. (2022a) also showed a spike-based framework combined with the entropy theory, called heterogeneous ensemble-based spike-driven few-shot online learning (HESFOL), that allows the neural network to learn fast on a few shots for diverse purposes. Another work from Yang et al. (2022b) used the entropy theory and recurrent spike neural networks to shape a spike-based framework with minimum error entropy, called MeMEE, and provided meta-learning capability for navigation, and another work, inspired by the functional neuroanatomy of the Basal Ganglia, (Yang et al., 2023b), pointed at higher performances on smart traffic navigation, Internet of Vehicles, based on a neuromorphic approach in a scalable and fault-tolerant framework. Our work does not lie on spiking neuron models, as the mentioned works do, but highlights the essential dynamics supported by neural ring attractors, as a novel neural structure for driving social navigation for robots.

Robots are also becoming increasingly prevalent in society, and it is likely that they will become even more important in the future. Research on social robots has been intensifying in recent years as more people use robots as companions, tools, and even as potential therapeutic agents (Sheridan, 2020; Jang et al., 2022). Social robots are utilized in different contexts, from healthcare settings to education, to entertainment, and even as personal companions (Hepp, 2020; Ozturkcan and Merdin-Uygur, 2022). Researchers have also explored how people interact with and respond to social robots, and how robots can be designed to be more human-like (Breazeal et al., 2008; Chaves and Gerosa, 2021; Tuli et al., 2021).

Furthermore, the use of robots as guides in unknown places has been studied in order to, for instance, help visitors explore their surroundings (Capi et al., 2014; Parra et al., 2019). This type of mobile robot guide has been designed to provide safety and security in unfamiliar places. Robot guides can also be used to help visitors find their way around such as in museums, airports, and other public spaces (Velentza et al., 2020; Gasteiger et al., 2021), providing visitors with directions and other information such as points of interest (Wang and Christensen, 2018). As technology advances, more applications of social robots as guides in unknown places are likely to be developed. One important aspect of constructing social robots is being able to perform more effectively the aforementioned. Bio-inspired navigation (Gul et al., 2019; Mao et al., 2020), control (Pardo-Cabrera et al., 2022; Guerrero-Criollo et al., 2023), and sensing (Jovanović et al., 2019) are still in their early stages, but it is likely to become an increasingly significant part of robot technology in order to build more effective and efficient robots.

The Social Force Model (SFM) is a computational model used in crowd dynamics and pedestrian behavior analysis. Its objective is to simulate the movement of individuals in a crowd considering the social forces that influence their behavior. SFM is based on the principle that individuals in a crowd are subject to both physical forces (e.g., collision avoidance) and social forces (e.g., social norms, personal space). In SFM, each individual or agent is represented as a point mass with a set of forces acting on them. These forces include an attractive force toward the agent's desired destination, a repulsive force from other agents to avoid collisions, and other social forces that influence behavior in specific situations. By integrating these forces, SFM can simulate the collective movement of individuals in a crowd and provide insight into crowd dynamics.

Truong and Ngo (2017) developed a proactive social motion mode (PSMM) for socially aware robot navigation in dynamic and crowded environments. Their approach takes into account the social norms and conventions that govern human-robot interactions in crowded spaces and uses them to predict the intentions and behaviors of pedestrians. This allows the robot to plan its trajectory and movements in a socially acceptable manner while avoiding collisions and achieving its navigation goals. The PSMM is shown to be effective through simulation and real-world experiments, enabling the robot to navigate safely and proactively plans its trajectory with socially acceptable behaviors. Kivrak et al. (2021) describe a framework for mobile service robots that prioritizes human safety and comfort while navigating in low to average density environments. This approach uses a Collision Prediction based Social Force Model (CP-SFM) to generate human-friendly routes. Their model is enhanced through multi-level mapping, obstacle repulsion points, and CP-SFM for motion planning. The framework is implemented through Robot Operating System (ROS) nodes and successfully tested in both real-world and simulation environments.

In contrast to the Social Force Model that considers physical and social forces in crowd dynamics, our approach focuses on representing obstacles, whether they are social or non-social, as factors influencing the robot's movement. We employ a connectivity mechanism based on excitation vs. inhibition to establish a relationship between the target position and the presence of obstacles. This connectivity determines the allowed directions for the robot to move, enabling it to navigate in a socially acceptable manner while avoiding collisions and reaching its desired destination.

Here, we propose a modular and adaptable approach for performing reactive navigation, allowing a robot to navigate through static or moving obstacles by computing competition, through ring attractor networks, between target and obstacle positions projected in the network. Our approach acts as a framework for organizing and processing sensory information, enabling efficient and effective navigation. We also compare our approach with existing navigation techniques to demonstrate its advantages and effectiveness.

## 2. Materials and methods

### 2.1. Software

#### 2.1.1. Python package

**Figure 1:**
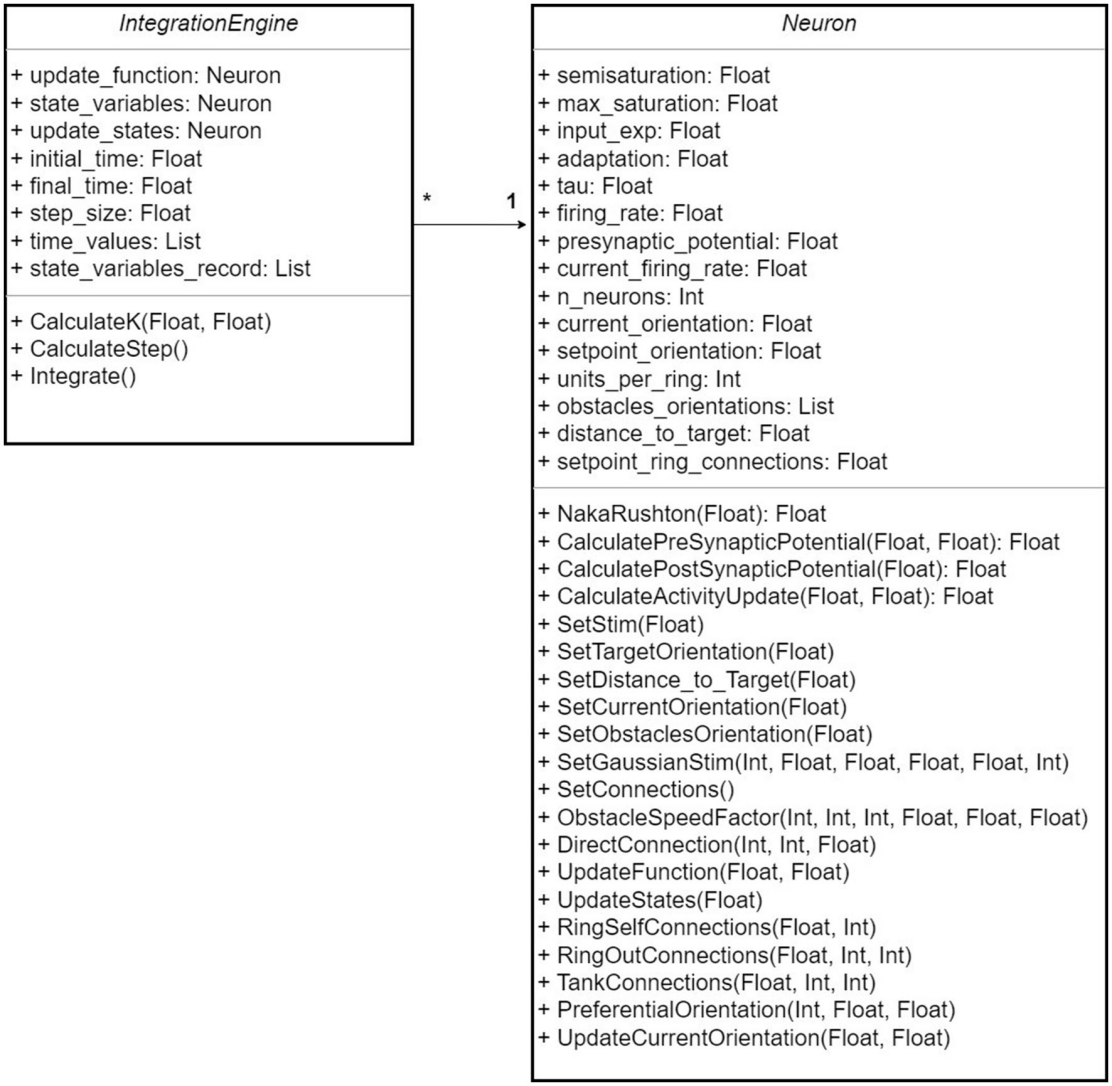
UML diagram for the proposed framework for the neural network design and simulation. “*” means the cardinality of the relationship between both classes.

We created a framework that separates the network structure from the integration mechanism, which allows us to make changes in both the neural network and the integration method independently. This framework is composed of two classes, the Neuron class in which the architecture and hyper-parameters of the network are defined and the IntegrationEngine class in which the integration mechanism is set. Currently, only the Runge-Kutta 4 method has been implemented. The UML diagram in Figure 1 shows the functions of each class. This framework was implemented in Python and makes use of the Numpy and Matplotlib libraries. Our work has been made public in GitHub, see Rivero-Ortega et al. (2022).

#### 2.1.2. ROS package

**Figure 2:**
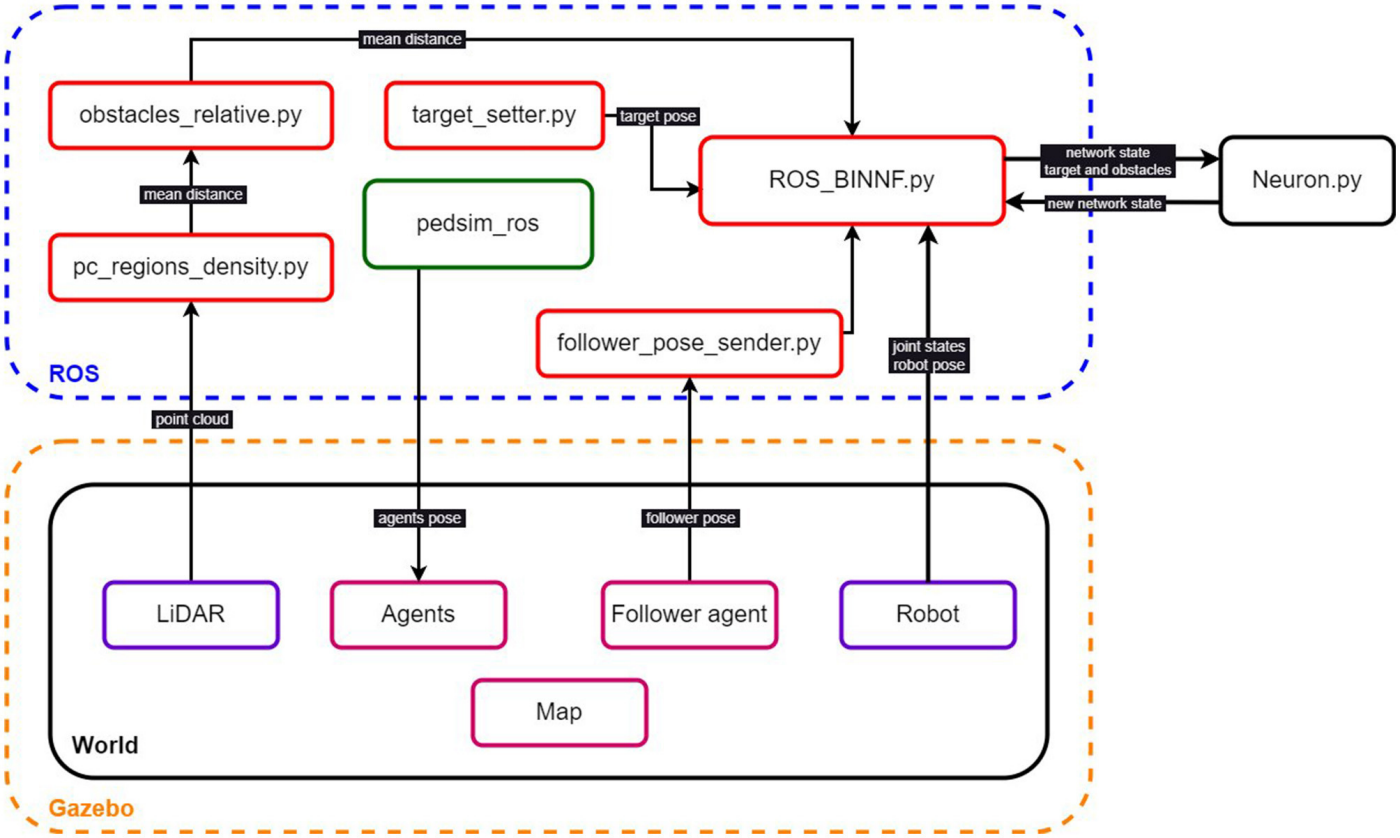
System diagram. ROS nodes in the blue dashed box. The Gazebo environment in the orange dashed box. Red boxes represent nodes that correspond to our proposed system. The green box corresponds to the PedSim ROS package. The magenta boxes refer to social agents and environment obstacles. The violet boxes represent the robot's information about the environment (laser and odometry data).

To enable our neural network to control a robot interacting with a virtual environment, we adapted the IntegrationEngine class as a ROS node. The ROS version was Noetic on Ubuntu 20.04.5 LTS. The virtual simulations were performed on Gazebo 11. The system diagram is shown in Figure 2, where the different nodes and subsystems are depicted. The ROS_BINNF node performs the integration of the dynamical system that represents the neural network defined in Neuron.py. LiDAR information passes through the pc_regions_density node where it is clustered along 24 directions around the robot, resulting in the mean distance to obstacles in a certain direction. Then this is converted from an absolute to a relative coordinate system to then feed the obstacles-related information in the ROS_BINNF node. The PedSim ROS package (Okal and Arras, 2014) simulates the social agents in the simulation and sends this data for Gazebo to visualize them. As the follower agent can be simulated with or without PedSim ROS, he/she is not connected in the diagram. The target position is set in the target_setter node. In each integration step, the ROS_BINNF node sends information about the obstacles, target, and current state of the robot, along with the last step neural network's states to the Neuron class.

### 2.2. Hardware

Our work points to evaluating the effectiveness of our neural network in controlling a robot's movement in environments with obstacles while guiding a social agent to a target position. To achieve this goal, we conducted experiments using two distinct setups. In Setup 1, the position of social agents in the environment was directly extracted from PedSim. In contrast, Setup 2 involved a more realistic simulation, where the position of social agents was inferred from a simulated velodyne LiDAR in Gazebo. Setup 1 was composed of an Intel Core i7-8700 CPU with a UHD Graphics 630 GPU and 16 GB RAM. On the other hand, Setup 2 was composed of an AMD Ryzen 9 5900HS, NVIDIA RTX 3,060 Mobile GPU, and 16 GB RAM.

### 2.3. Modeling

The neural network in our work is based on the Wilson-Cowan model, which utilizes a differential equations system to model the behavior of neurons. Specifically, a given neuron is represented by Equation (1), where *Y* denotes the activity level of the unit, τ represents the time constant, and *Z* represents the net input received by the neuron. The function *f*(*Z*), named the Naka-Rushton function and described in Equation (2), serves as the activation function for all neurons within the network. This function is characterized by three parameters: γ, σ, and μ. Specifically, γ represents the maximum output value of a neuron, σ represents the input level at which the neuron outputs half its maximum value, and μ controls the slope of the function curve.


(1) $$\frac{\text{d}Y}{\text{d}t}=\frac{1}{\tau }\left[-Y+f\left(Z\right)\right]$$

(2) $$f\left(Z\right)=\{\begin{array}{ll} \gamma \left(\frac{Z^{\mu }}{\sigma^{\mu }+Z^{\mu }}\right) & \text{if Z}\geq \text{0},\\ 0 & \text{if Z}<\text{0}. \end{array}$$

All neurons in the proposed network share identical values for the Naka-Rushton function parameters and the time constant. The precise values of the parameters of the proposed network are compiled in Table 1. These parameters were established by trial and error in order to obtain the expected behavior. These tests did not take into account sensory loss. It served as a proof of concept step to perform a fine tuning process through the parameter sweep shown in Section 2.3.7.

**Table 1:** Network parameters.

**Parameter**	**Value**
τ	0.001
γ	100.0
μ	2
α	5.0
σ	40.0
ξ	1.2
η	0.0005
*c*	120.0
ω^*CA*^	2.0
ω^*CB*^	2.0
ω^*ED*^	1.0
ω^*FC*^	1.0
ω^*GE*^	1.0
ω^*HF*^	1.0
ω^*IG*^	1.0
ω^*IH*^	1.0
ω^*JH*^	1.0
ω^*JG*^	1.0
ω^*KI*^	0.75
ω^*KJ*^	0.75
ω_*U*_	0.5
ω_*V*_	1.0
ω_*S*_	0.0125
ω^*EC*^	1.0
ω^*ED*^	1.0
ω^*FD*^	1.0
$\omega_{j}^{CC}$	$\begin{array}{l} \;\omega_{j}^{CC}=1.9for\;a\;ll\;1<=j<4\\ \omega_{j}^{CC}=-1.7for\;all\;4<=j<=24 \end{array}$
$\omega_{j}^{DD}$	$\begin{array}{l} \;\omega_{j}^{DD}=0.8for\;a\;ll\;1<=j<4\\ \omega_{j}^{DD}=-0.9for\;all\;4<=j<=24 \end{array}$

The proposed neural network consists of six ring-shaped layers and a motor command neural circuit, each with a specific purpose. The first layer, “the target encoding layer,” encodes the orientation of the robot's target position. The second layer, “the obstacles encoding layer,” encodes the angular position of obstacles detected by the robot. The third layer, “the setpoint encoding layer,” selects the direction in which the robot is allowed to move. The fourth layer, “the robot orientation encoding layer,” encodes the current orientation of the robot, and the fifth and sixth layers, “the error encoding layers,” compute the error between the target and current orientation. Each layer is composed of 24 units.

#### 2.3.1. Target encoding layer

The target encoding layer is responsible for representing the desired orientation of the robot's movement. This is achieved by projecting a Gaussian-shaped activity bump on the layer, centered around the target orientation θ_*T*_. The target orientation depends on the position of the robot at any given time. Each unit in this layer, denoted as *A*_*i*_, has a preferred direction Ω_*i*_ and is described by the activity level *A*, which is governed by Equation (3). The parameter α represents the minimum activity level, while γ represents the maximum activity level of the neuron. The time constant τ determines the rate at which the activity level changes. The width of the bump is given by the parameter ξ, which controls the selectivity of the neurons to the target angle.


(3) $$\begin{array}{l} \frac{\text{d}A_{i}}{\text{d}t}=\frac{1}{\tau }\left[-A_{i}+f\left(\alpha +\left(\gamma -\alpha \right)e^{-\frac{{\left({\text{Ω}}_{i}-\theta_{T}\right)}^{2}}{2\xi^{2}}}\right)\right] \end{array}$$

#### 2.3.2. Obstacles encoding layer

In this layer, the obstacles are encoded based on the presence of obstacles in a particular direction. Higher activity in a unit means there are obstacles near to the robot in a particular direction and lower activity means the opposite. The dynamic of neuron *i* in layer *B* is represented by Equation (4). The variable ρ_*i*_ denotes the distance from the robot to an obstacle in the direction of Ω_*i*_, the preferred direction of neuron *B*_*i*_. To map the distance to an activity level input for the layer, we use a distance function defined by Equation (5).


(4) $$\begin{array}{l} \frac{\text{d}B_{i}}{\text{d}t}=\frac{1}{\tau }\left[-B_{i}+f\left(g\left(\rho_{i}\right)\right)\right] \end{array}$$

The distance function, Equation (5), is defined as a piece-wise function that maps the distance to the corresponding activity level for the neuron. The activity level is given by *c*/ρ_*i*_ where *c* is a scaling factor that determines how sensitive the neuron is to changes in the distance to an obstacle. A larger value of *c* results in a steeper increase in activity level as the distance decreases, while a smaller value of *c* results in a more gradual increase. Because the maximum activity level of a neuron in our network is γ, if the value of *c*/ρ_*i*_ surpasses that level, the function's output becomes γ.


(5) $$g(\rho_{i})=\{\begin{array}{ll} \gamma , & \text{if }\frac{c}{\rho_{i}}\geq \gamma \\ \frac{c}{\rho_{i}}, & \text{if }\frac{c}{\rho_{i}}<\gamma \end{array}$$

#### 2.3.3. Setpoint encoding layer

**Figure 3:**
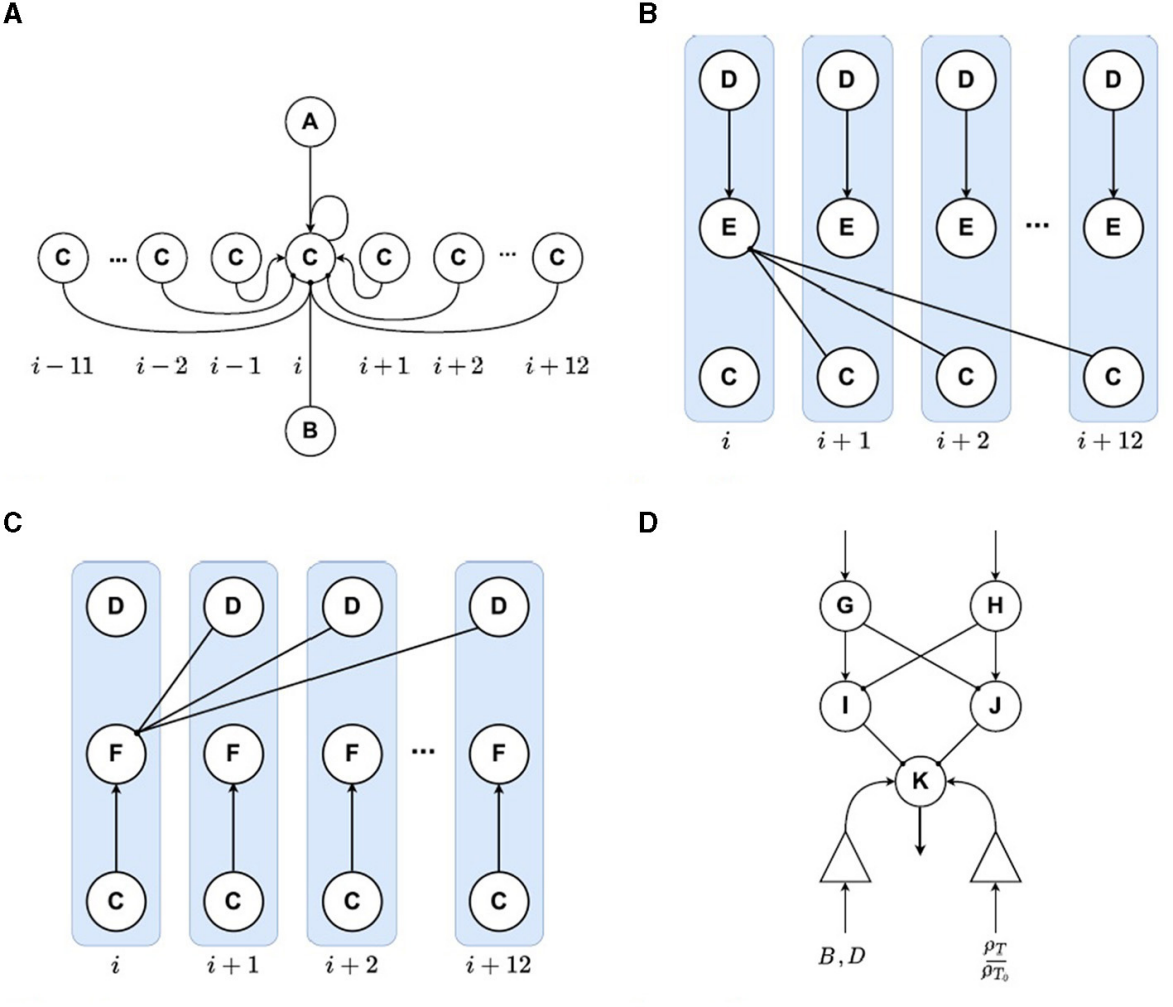
Connectivity diagrams for different layers of the proposed model. Inhibitory connections are represented by circled endings and excitatory connections are represented by arrow endings. **(A)** WTA connectivity. **(B, C)** Connections for the layers that encode the error for clockwise and counter-clockwise turns. **(D)** Connections for the motor command circuit.

The setpoint encoding layer plays a crucial role in determining the robot's direction of movement. Firstly, this layer computes a subtraction operation between the output of units encoding the target orientation and units encoding the obstacles thus producing a high activity level in directions for which there are no obstacles. This subtraction operation is evident in Equation (6) where there are somatotopic excitatory connections from the target encoding layer (*A* units) and somatotopic inhibitory connections from the obstacles encoding layer (*B* units). Secondly, intra-layer connections (shown in Figure 3A) that correspond to the winner-take-all (WTA) structure, facilitate competition between the layer's units. This competition selects only a limited number of permitted directions for the robot to move toward.


(6) $$\begin{array}{l} \frac{\text{d}C_{i}}{\text{d}t}=\frac{1}{\tau }\left[-C_{i}+f\left(\omega^{CA}A_{i}-\omega^{CB}B_{i}+\overset{24}{\underset{j=1}{\sum }}\omega_{j}^{CC}C_{\left(j+i-3\right)\text{mod}24+1}\right)\right] \end{array}$$

The use of the modulus operator (mod24) ensures that the indices of the neighboring neurons wrap around the ring of 24 units.

#### 2.3.4. Robot orientation encoding layer

This layer is responsible for encoding the orientation of the robot by projecting sensor measurements onto neurons that are tuned to preferred orientations Ω_*i*_, which is represented by Equation (7). Here, Ω_*i*_ denotes the preferred direction of unit *i*, while θ_*r*_ corresponds to the orientation of the robot at any given time. The activity level of neuron *i* is represented by *D*, and the self-connections in the layer are described by the weights ω^*D*^, that connect neuron *i* and *j*mod24. Due to these self-connections, this layer expresses a WTA structure similar to the setpoint encoding layer.


(7) $$\begin{array}{l} \frac{\text{d}D_{i}}{\text{d}t}=\frac{1}{\tau }\left[-D_{i}+f\left(\gamma \cos\left({\text{Ω}}_{i}-\theta_{r}\right)+\overset{24}{\underset{j=1}{\sum }}\omega_{j}^{DD}D_{\left(j+i-3\right)\text{mod}24+1}\right)\right] \end{array}$$

The term, γcos(Ω_*i*_−θ_*r*_), measures the similarity between the preferred orientation of neuron *i* and the current orientation of the robot, and it determines the activity level of the neurons in this layer. The self-connections in this layer allow competition between neurons that respond to different preferred orientations, resulting in the selection of a reduced group of neurons whose activity level represents the current orientation of the robot.

#### 2.3.5. Error encoding layers

As mentioned before, the neural network includes two orientation error encoding layers that measure the distance between the current orientation and the target orientation. The first of these two layers, consisting of units labeled *E*, encodes errors for clockwise rotations, while the second layer, consisting of units labeled *F*, encodes errors for counterclockwise rotations. To achieve this, *E* receives excitatory one-to-one projections from the current orientation layer and inhibitory connections from units to its right in the setpoint layer, see Figure 3B. In contrast, *F* receives one-to-one excitatory connections from the setpoint layer and inhibitory connections from the current orientation layer to its right, see Figure 3C. The clockwise error encoding layer is described by Equation (8), in which *E* is the activity level for neuron *i* in the layer, and $\omega_{j\text{mod}24}^{EC}$ is the weight that connects neuron *i* in layer *E* with neuron *j*mod24 in layer *C*. Additionally, ω^*ED*^ is the weight of the connection coming from neuron *i* in layer *D* to its counterpart in layer *E*.


(8) $$\begin{array}{l} \frac{\text{d}E_{i}}{\text{d}t}=\frac{1}{\tau }\left[-E_{i}+f\left(\omega^{ED}D_{i}-\overset{i+11}{\underset{j=i}{\sum }}\omega^{EC}C_{j\text{mod}24+1}\right)\right] \end{array}$$

The counterclockwise error encoding layer is defined analogously to its clockwise counterpart, as expressed in Equation (9). The neural activity level for neuron *i* in this layer is denoted by *F*_*i*_, and the weight of the connection between neuron *i* in layer *F* and neuron *j*mod24 in layer *D* is represented by $\omega_{j\text{mod}24}^{FD}$. The weight of the connection from unit *i* in layer *D* to the corresponding unit in layer *F* is denoted by ω^*FC*^.


(9) $$\begin{array}{l} \frac{\text{d}F_{i}}{\text{d}t}=\frac{1}{\tau }\left[-F_{i}+f\left(\omega^{FC}C_{i}-\overset{i+11}{\underset{j=i}{\sum }}\omega^{FD}D_{j\text{mod}24+1}\right)\right] \end{array}$$

#### 2.3.6. Motor command circuit

The motor command circuit is a neural circuit that generates motor commands for the robot. It is composed of five neurons, two of which, designated as *G* and *H*, act as accumulation units. These neurons integrate the activity from the clockwise and counterclockwise error encoding layers and indicate the appropriate direction for the robot to rotate to achieve the desired outcome. Neuron *I* receives excitatory projections from neuron *G* and inhibitory projections from neuron *H*, while the opposite is true for neuron *J*, which receives excitatory projections from neuron *H* and inhibitory projections from neuron *G*. The dynamics of neurons *G*, *H*, *I*, and *J* are governed by Equations. (10)–(13).


(10) $$\begin{array}{l} \frac{\text{d}G}{\text{d}t}=\frac{1}{\tau }\left[-G+f\left(\overset{24}{\underset{j=1}{\sum }}\omega^{GE}E_{j}\right)\right] \end{array}$$

(11) $$\begin{array}{l} \frac{\text{d}H}{\text{d}t}=\frac{1}{\tau }\left[-H+f\left(\overset{24}{\underset{j=1}{\sum }}\omega^{HF}F_{j}\right)\right] \end{array}$$

(12) $$\begin{array}{l} \frac{\text{d}I}{\text{d}t}=\frac{1}{\tau }\left[-I+f\left(\omega^{IG}G-\omega^{IH}H\right)\right] \end{array}$$

(13) $$\begin{array}{l} \frac{\text{d}J}{\text{d}t}=\frac{1}{\tau }\left[-J+f\left(\omega^{JH}H-\omega^{JG}G\right)\right] \end{array}$$

The generation of forward motion in the robot is controlled by the dynamics of neuron *K*, as described by Equation (14), which receives input from three sources. First, the activity from neurons *I* and *J* is integrated via inhibitory connections. Second, the distance between the robot's current position and the target position is considered, resulting in an increase in speed as the robot moves further away from the target, as indicated by the ratio $\frac{\rho_{T}}{\rho_{T_{0}}}$. Lastly, the activity of the obstacles and robot orientation layers are multiplied and summed to determine if there are obstacles in the robot's path, leading to a decrease in speed if any obstacles are present in the direction the robot is moving toward. This interaction is formulated by Equation (15). Figure 3D shows the circuit structure.


(14) $$\begin{array}{l} \frac{\text{d}K}{\text{d}t}=\frac{1}{\tau }\left[-K+f\left(-h\left(B,D\right)-\omega^{KI}I-\omega^{KJ}J+\gamma \frac{\rho_{T}}{\rho_{T_{0}}}\right)\right] \end{array}$$

(15) $$h\left(U,V\right)=\sum_{j=1}^{24}\frac{\omega_{U}U_{j}\;\omega_{V}V_{j}}{\omega_{S}}$$

#### 2.3.7. Parameter sweep

We performed a parameter sweep for the synaptic weights of the attractor rings that encode the setpoint orientation and the current orientation. The remaining weights stayed as in the proof of concept since the required function was based on the model architecture instead of the specific values of the weights. It is important that the parameters of the attractor rings allowed not only a bump in activity while there was sensory input, but also when there was an absence of sensory input. Therefore, the parameter sweep was performed in two scenarios, the first one in which sensory input is maintained and another in which it is removed after some time. All the synaptic weights of the inhibitory connections of both rings were varied jointly, as well as the excitatory connections. The scanning was performed in the range of 0.1 to 1.95 in steps of 0.05.

The expected behavior of both rings was that only three units would be active in the scenarios mentioned above. Such behavior was achieved in the setpoint ring when the inhibitory weights were 0.60 and in the current orientation ring when the inhibitory weights were equal to or greater than 0.55. Sustained activity after the removal of sensory input became evident when the excitatory weights of both rings were 0.4. Also, the values for which the excitatory weights allowed only three active units depended on the value of the inhibitory weights, resulting in a large number of operating windows for the model. The results for the parameter sweep are registered as videos and will be provided as Supplementary material.

### 2.4. Social navigation metrics

There are several metrics that can be employed to assess the effectiveness of social robot navigation in terms of human acceptance. These metrics may include various indicators that aim to ensure people's comfort and ease, and we use the following.

The Social Individual Index (SII) was introduced by Truong and Ngo (2017) as a metric for establishing a safe distance between a social robot and an individual. The SII is calculated using Equation (16), where ($x_{i}^{p}$, $y_{i}^{p}$) represents the position of the human and (*x*_*r*_, *y*_*r*_) represents the position of the robot. The parameter $\sigma_{0}^{p}$ is set to $\frac{d_{c}}{2}$, where *d*_*c*_ is typically between 0.45 m and 1.2 m in accordance with Hall's personal space (Hall, 1966), and *N* represents the number of humans located near the robot.


(16) $$\begin{array}{l} \text{SII}=\underset{i=1:N}{\max}exp\left(-\left({\left(\frac{x_{r}-x_{i}^{p}}{\sqrt{2}\sigma_{0}^{p}}\right)}^{2}+{\left(\frac{y_{r}-y_{i}^{p}}{\sqrt{2}\sigma_{0}^{p}}\right)}^{2}\right)\right) \end{array}$$

The Relative Motion Index (RMI) is a metric developed by Truong and Ngo (2017) that estimates the probability of a collision between a social robot and an individual. This metric takes into account the speed and direction of both the robot and people, and it is most significant when a robot and a human are approaching each other at their maximum speeds. The RMI is calculated using Equation (17), where $v_{i}^{p}$ and *v*_*r*_ represent the velocities of the person *p*_*i*_ and the robot, respectively. The parameter β_*i*_ represents the angle between the robot orientation and the vector projected from the robot to the human *pi*, while φ_*i*_ represents the angle between the person orientation and the vector projected from the person to the robot.


(17) $$\begin{array}{l} \text{RMI}=\underset{i=1:N}{\max}\frac{2+v_{r}\cos\left(\beta_{i}\right)+v_{i}^{p}\cos\left(\varphi_{i}\right)}{\sqrt{{\left(x_{i}^{p}-x_{r}\right)}^{2}+{\left(y_{i}^{p}-y_{r}\right)}^{2}}} \end{array}$$

### 2.5. Framework structure

The navigation system is divided into perception, planning, and control stages (as proposed in our previous work Silva et al., 2022, 2023). In the perception stage, data from the environment is obtained and processed. In the planning stage, the neural network is supplied with information about the environment and the goal position and generates control commands for the robot as output from the neural network. In the control stage, the signals coming from the network are decoded to determine the velocities for the differential control of the robot.

The velocities set in the control stage are conditioned by the distance to the user, the distance to the target position, and the SII and RMI values. The robot slows down when it moves away from the user, when it is close to the target position, or when it calculates high SII and RMI values.

### 2.6. Virtual environment

**Figure 4:**
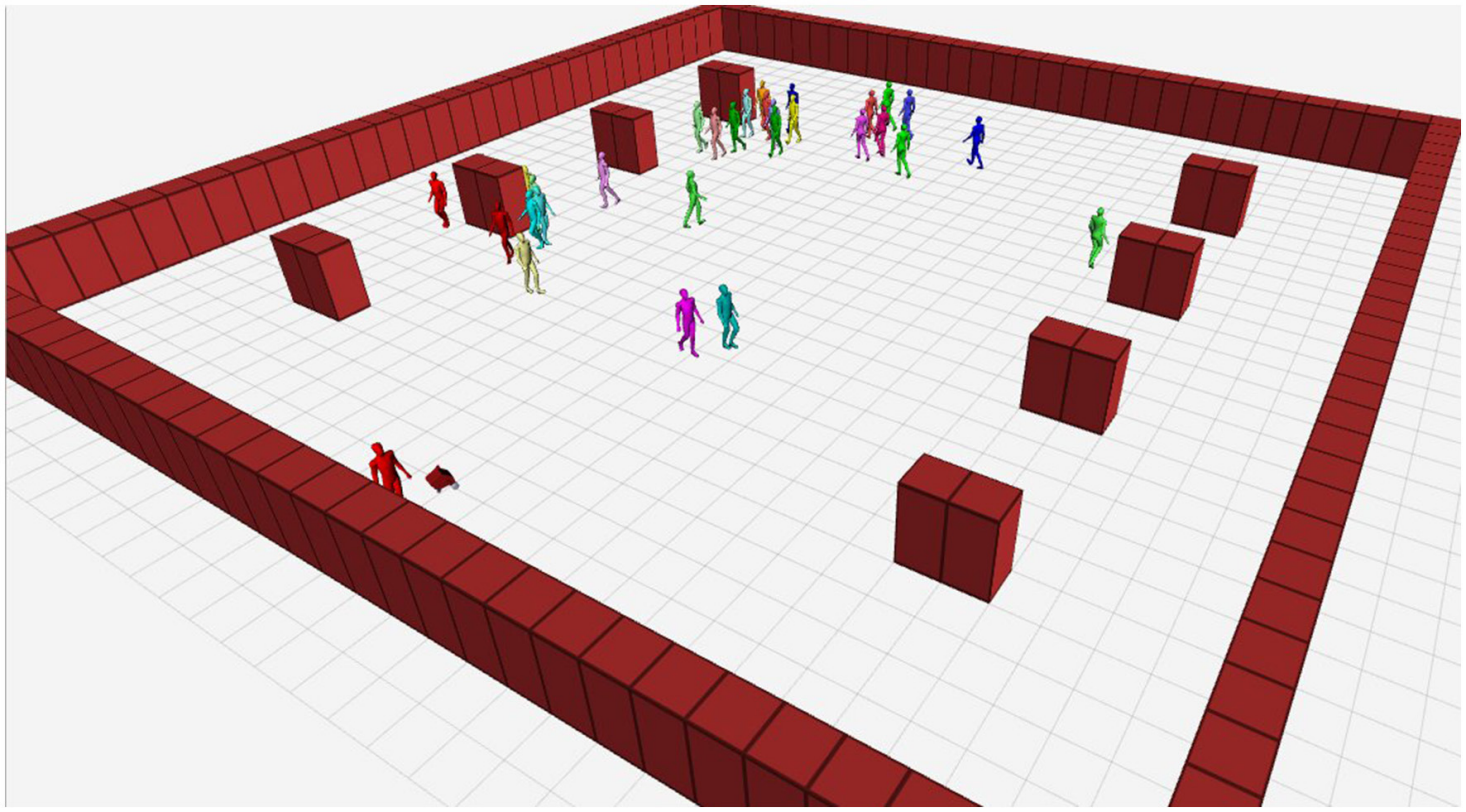
Virtual scenario. Visual representation in Rviz of static obstacles, dynamic agents, and the robot.

We simulated our experiments using Gazebo and a modified version of the pedestrian simulator, pedsim_ros (Okal and Linder, 2021), which is a set of ROS packages that provide a 2D pedestrian simulator based on the social force model. We created a virtual scenario of a pedestrian area with various obstacles and dynamic agents that resembled university campus areas with pedestrians moving in different directions according to predefined waypoints. To illustrate the scene, we present a visualization in Rviz in Figure 4, where the agent closest to the robot represents the guided person. We modified the pedsim_ros packages to turn the robot into an attractor waypoint for this agent.

The robot used in our simulation is a virtual model developed by students at the Universidad Autónoma de Occidente. The robot's description package is accessible through this link. It is equipped with a perception system that includes a camera and a LiDAR 3D sensor. The robot's locomotion system is based on a differential drive with two non-motorized castor wheels at the front and two standard fixed wheels at the rear.

## 3. Results

### 3.1. Neural network

After performing the parameter sweep explained in Section 2.3.7 we were able to get the model to perform as expected. We tested the model in different conditions of target, obstacles and initial orientation using the Python Package implementation.

#### 3.1.1. Ring attractor behavior

**Figure 5:**
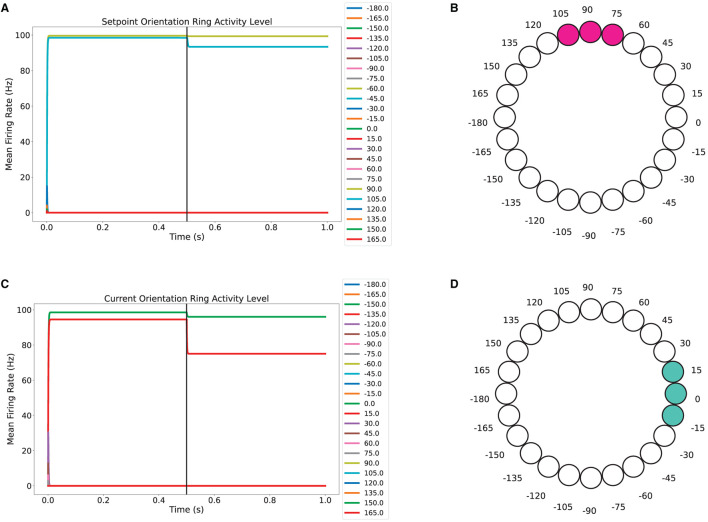
Stable dynamics of the ring attractor network for the setpoint orientation and current orientation rings. **(A, C)** Activity level of neurons over time for the setpoint orientation and current orientation rings. **(B, D)** Three neuron bump for encoding the 90° orientation of the target and the 0° for the current orientation.

To make sure that the attractor ring layers show the characteristics of this type of network, we tested different scenarios. An important feature to test was that in the absence of sensory input, the activity bump along the ring is preserved. The first scenario was stopping, at 0.5 seconds, the update of the target and the current orientation information to check the evolution of the activity level in the Setpoint Orientation and the Current Orientation layers. As shown in Figure 5A, the level of activity of the neurons in the setpoint orientation layer undergoes a small decrease when the target-related information is removed. However, from Figure 5B it can be noticed that the bump is preserved. A similar behavior is observed for the current orientation layer, as shown in Figures 5C, D.

#### 3.1.2. Re-orientation task in an unobstructed environment

**Figure 6:**
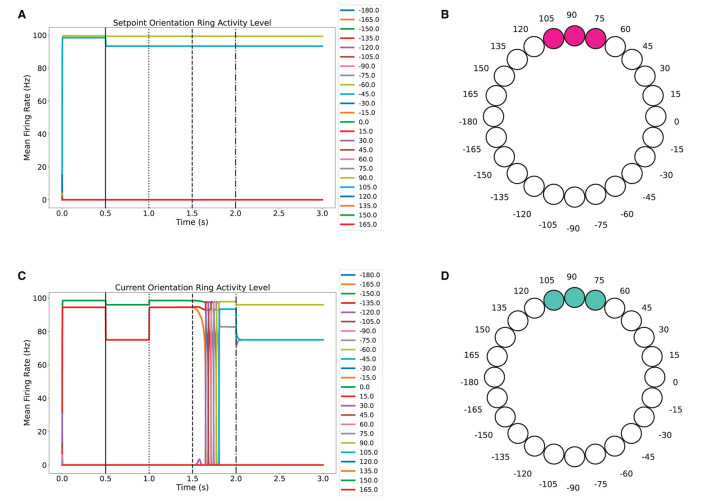
Re-orientation task in which an agent must rotate from **0°** to **90°** in an unobstructed environment. **(A, C)** Activity level of neurons over time for the setpoint orientation and current orientation rings. **(B, D)** Three neuron bump for encoding the 90° orientation of the target and the final 90° current orientation.

The next step was testing the model's ability to direct the orientation of a simulated agent toward a specified target direction in an unobstructed environment. In this experiment, the sensory input for both the target and current orientations was enabled for the first 0.5 seconds. After that, the sensory input was disabled for both of them, as can be seen in Figures 6A, C, where the activity level decreases for the following 0.5 seconds. At the first second, the sensory input for the current orientation ring was restored by decoding the orientation using Equation (18). In this equation, *y* corresponds to the activity level for neuron *i* in the current orientation ring, and Ω is the angle that is represented by neuron *i* in the current orientation ring.


(18) $$\text{decoded orientation}=\frac{\sum_{i}^{24}{\text{D}}_{i}{\text{Ω}}_{i}}{\sum_{i}^{24}D_{i}}$$

After updating the current orientation input, the system is allowed to re-orient itself at *t* = 1.5 seconds. This is done by updating the current orientation with Equation (19), where *G* and *H* are the activity levels of the accumulation units that promote clockwise and counter-clockwise movement, respectively. The parameter η controls how aggressive the orientation update is. Finally, at *t* = 2.0 seconds, the sensory input is disabled again for the current orientation ring. As shown in Figure 6D, the final current orientation matches the desired target orientation, which is around 90°, as shown in Figure 6B.


(19) $$\begin{array}{l} \text{current orientation}=\text{current orientation}+\eta \left(G-H\right) \end{array}$$

#### 3.1.3. Re-orientation task in an obstructed environment

**Figure 7:**
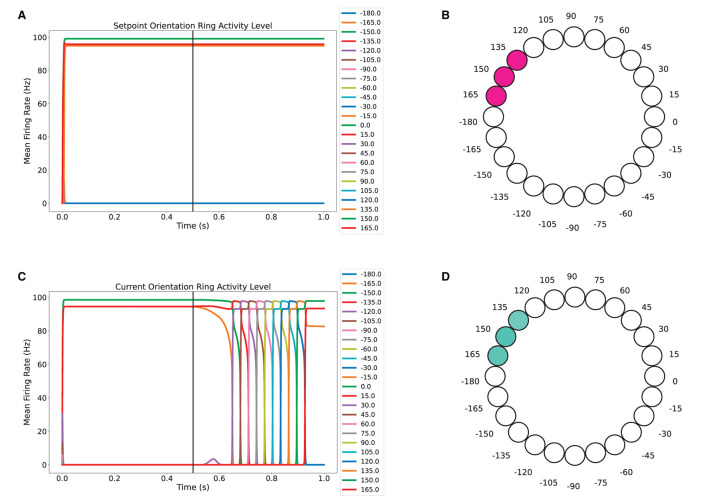
Re-orientation task in an obstructed environment. The obstacles match the 90° orientation of the target. **(A, C)** Activity level of neurons over time for the setpoint orientation and current orientation rings. **(B)** Three neuron bump for encoding the estimated allowed orientation in the setpoint ring. **(D)** Three neuron bump for encoding the final orientation of the agent after re-orienting itself.

Finally, the model was tested in a scenario in which there is an obstacle in the same direction in which the robot is heading, which is 90°. As noticed in Figure 7A, the sensory input is maintained along the simulation. Because the target orientation and the obstacles orientation coincided, the setpoint orientation found an allowed direction of movement toward 150°, see Figure 7B. In this experiment, the agent was allowed to re-orient at *t* = 0.5 seconds, as shown in Figure 7C, when the activity level of neurons in the current orientation ring changed. As evident in Figure 7D, the agent was able to re-orient to match the setpoint orientation.

### 3.2. Virtual robot simulation

The introduced bio-inspired navigation system enabled a robot to guide a social agent (person) to a specific destination while avoiding both static and dynamic obstacles. Our system utilized a novel architecture that integrates sensory representation, decision-making, and motor control through a connectivity pattern between two ring attractor networks. To evaluate the performance of our system, we compared it with the widely used Social Force Model and the Optimal Rapidly-exploring Random Tree (RRT*) planning algorithm (Karaman and Frazzoli, 2011), using the Social Individual Index and the Relative Motion Index as metrics for comparison.

The RRT* algorithm was implemented using the Open Motion Planning Library (OMPL) (Şucan et al., 2012). In the collision evaluation phase, a circular area with a radius of 1 meter was defined around the social agents (except for the user to be guided), so that the robot took into account the personal space during navigation. Since this is an environment with dynamic obstacles, path planning was performed with an update period of 1 second.

In order to obtain a socially acceptable behavior, paths were planned using Dubins curves that allowed straight-forward moves and right or left turns (as proposed in our previous work Hernández et al., 2016, 2019). To execute the planned paths, the angular and linear velocity of the robot were set to 1 m/s.

The algorithms were compared in a virtual scenario simulated in Gazebo. The behavior of the agents was generated using a customized version of pedsim_ros, which allowed to represent the robot as an attractor node for the guided social agent, simulating the guiding task. The scenario consisted of a space of 30 × 30 meters with static obstacles inside and social agents. The agents started by locating themselves around 4 coordinates given in Table 2, and moved along a route defined by the associated waypoints.

**Table 2:** Number of agents in each scenario [low, medium, and high population], initial coordinates and paths (defined by waypoints) for each agent.

**Number of agents**	**Initial position (x, y)**	**Waypoints**
4, 10, 30	(6, 5)	1, 2, 3, 4
2, 5, 15	(7, 5)	2, 3, 4
4, 10, 30	(24, 25)	3, 4, 1, 2
2, 5, 15	(23, 25)	3, 2

**Figure 8:**
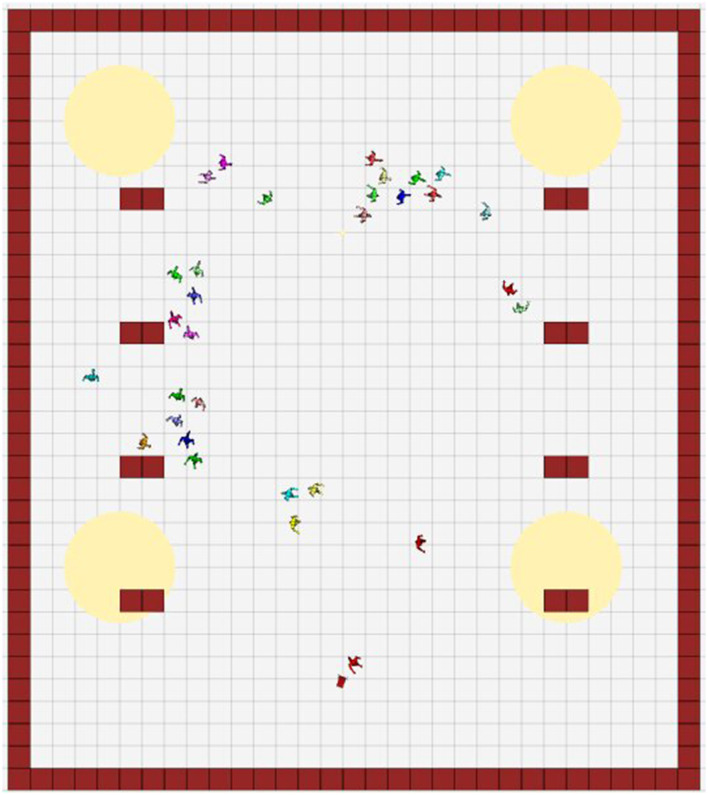
Virtual scene. Global view (Rviz). Waypoints (yellow circles), static obstacles (red squares), the robot (at the bottom), and social agents.

Three scenarios were simulated, maintaining the static structure of the environment and varying the number of social agents between 10, 30 and 90, showing low, medium and high populated scenarios. The waypoints (in pedsim_ros) corresponded to attractor nodes for the agents. A circular area with a radius of 5 meters was established around the waypoints, so that upon reaching this area, the agent was attracted to the next waypoint on its path. Table 3 and Figure 8 show the established waypoints.

**Table 3:** Waypoints locations (pedsim_ros).

**Waypoint**	**Coordinate (x, y)**
1	(25, 5)
2	(25, 25)
3	(5, 25)
4	(5, 5)

**Figure 9:**
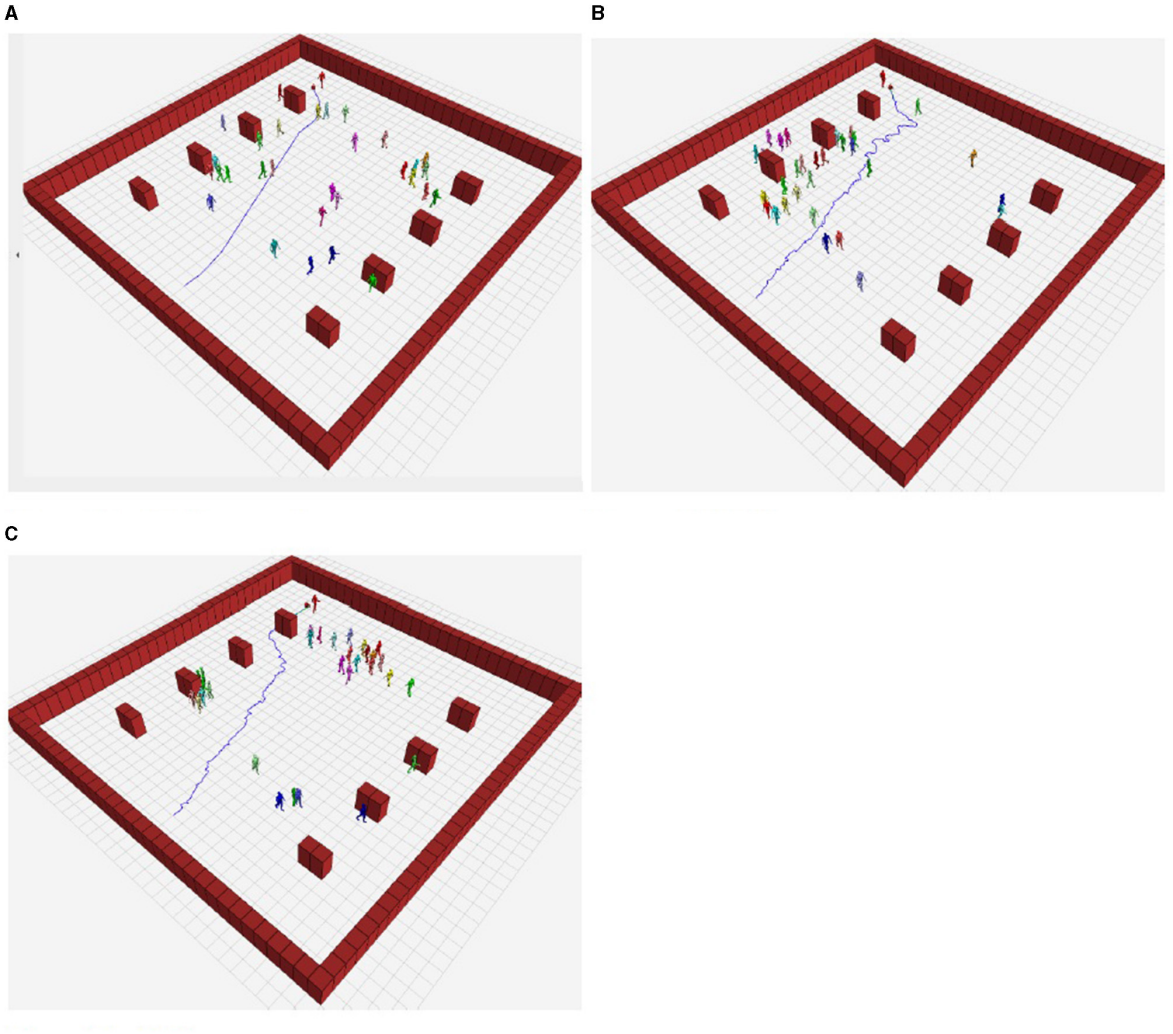
Robot path in each scenario for the same position with each algorithm. **(A)** Our framework. **(B)** RRT*. **(C)** SFM.

On the virtual stage, 100 random target positions (in collision-free space) were generated. The algorithms were tested at each position in all scenarios, with metrics recorded for each run. Table 4 shows the mean and standard deviation of the metrics obtained during the experiments, while Figure 9 shows the robot path in scenario 1 for the same target position using the compared algorithms.

**Table 4:** Metrics obtained with each approach.

	**Scenario 1**		**Scenario 2**		**Scenario 3**	
	**SII mean (std)**	**RMI mean (std)**	**SII mean (std)**	**RMI mean (std)**	**SII mean (std)**	**RMI mean (std)**
Our proposal	0.246 (0.022)	2.059 (0.394)	0.250 (0.034)	2.057 (0.427)	0.264 (0.118)	2.286 (0.360)
SFM	0.252 (0.028)	2.065 (0.401)	0.251 (0.050)	2.032 (0.455)	0.268 (0.126)	2.315 (0.402)
RRT*	0.255 (0.034)	2.061 (0.421)	0.259 (0.053)	2.107 (0.405)	0.263 (0.124)	2.297 (3.596)

## 4. Discussion

Overall, our proposed bio-inspired navigation system showed promising results in guiding a robot to a target location while avoiding obstacles. The integration of sensory representation, decision-making, and motor control through ring attractor networks offers a biologically-inspired approach for navigation in complex environments. These findings highlight the potential of our system for further applications in various robotic platforms and real-world scenarios.

The neural network features a unique integrated architecture with ring attractor networks. The ring attractor network provides a biologically inspired approach to navigation and enables the robot to adapt to changes in the environment. It is worth noting that all neurons in the proposed network share identical values for the activation function parameters and the time constant. This observation highlights the crucial role of the network architecture in computing an orientation error value that enables the control of both a robot's orientation and speed. The use of identical values ensures a consistent and coherent orientation control strategy, which is essential for successful navigation in dynamic environments.

SFM focuses on crowd behavior and social forces such as attraction, repulsion, and social norms and the proposed approach does not consider these forces; instead, it utilizes an attractor ring structure to encode the target and obstacles. Additionally, the proposed approach incorporates the Social Individual Index to establish a safe distance between the robot and an individual, and the Relative Motion Index to estimate collision probability based on relative speeds and directions.

Our approach points to improving safety and comfort specifically for human-robot interactions. By integrating SII and RMI, this approach considers both social comfort and collision avoidance features, resulting in better human-robot interactions in a crowded environment. The results obtained in our experiments demonstrate the effectiveness of this architecture in guiding a social agent while avoiding obstacles, and the metrics used for evaluating the system indicate that our proposal outperforms the widely used Social Force Model. The achievement of lower SII and RMI metrics than the widely used SFM allows the conclusion that our proposal can generate socially acceptable navigation with a lower probability of causing discomfort to the surrounding people.

Our proposed system, similar to the SFM, generates reactive navigation, which means that the robot's movements are determined in real-time based on interactions with the environment through sensors. Unlike other planners, such as grid-based path planners, our system does not rely on a prior description of the environment, which makes it impossible to guarantee that the navigated route is the most optimal. Instead, our system relies on the perception module to obtain information about the environment, including the presence of obstacles and social agents. It should be noted that the performance of our system may differ in real-world implementations where sensors are constrained or face limitations.

Sampling-based motion planning methods, such as RRT*, generate different random configurations in a free configuration space and determine the optimal path between the start and goal positions by evaluating the links between each configuration. In obstacle avoidance, reactive methods perform a trajectory modification when the robot is close to the obstacle. Predictive navigation conceives the avoidance, starts from planning, and evaluates the path in all generated configurations, which leads to higher computational cost and longer algorithm execution time. The computational cost increases when replanning in dynamic environments where obstacles change their position compared to previously planned paths. This, added to the planning time and the replanning period, leads to the execution of “discontinuous” routes, which may be perceived as socially unacceptable movements when navigating in the presence of people.

In future work, we plan to conduct real-life implementations to validate the simulation results, with a focus on social metrics that reflect people's comfort. Additionally, we intend to expand our consideration of social behaviors beyond those related to proxemics, such as the activity zone, which may affect human-robot interactions.

## Data availability statement

The datasets presented in this study can be found in online repositories. The names of the repository/repositories and accession number(s) can be found below: https://github.com/JesusRiveroOrtega/BINNF.git.

## Author contributions

JR-O designed, implemented, and tested the proposed model. JM-M designed and implemented the social context simulation and metrics, and collected, compiled, and analyzed the simulation results. JH supervised the work related to social robot navigation, including the social context, its simulation, and benchmarking of the selected social metrics. VR-C supervised the work related to the use of social robot navigation for guiding social agents. JH-L and JP-C reviewed the state of the art. DR-M proposed the research topic and was in charge of guiding the workflow. DR-M and JH-L reviewed and read-proofed the manuscript. All authors contributed to the writing, editing, and formatting of the manuscript and approved the submitted version.
