# Corrigendum: Ring attractor bio-inspired neural network for social robot navigation

**DOI:** 10.3389/fnbot.2023.1304597

**Published:** 2023-10-19

**Authors:** Jesús D. Rivero-Ortega, Juan S. Mosquera-Maturana, Josh Pardo-Cabrera, Julián Hurtado-López, Juan D. Hernández, Victor Romero-Cano, David F. Ramírez-Moreno

**Affiliations:** ^1^Department of Engineering, Universidad Autónoma de Occidente, Cali, Colombia; ^2^Department of Mathematics, Universidad Autónoma de Occidente, Cali, Colombia; ^3^School of Computer Science and Informatics, Cardiff University, Cardiff, United Kingdom; ^4^Robotics and Autonomous Systems Laboratory, Faculty of Engineering, Universidad Autonoma de Occidente, Cali, Colombia; ^5^Rimac Technology, Zagreb, Croatia; ^6^Department of Physics, Universidad Autónoma de Occidente, Cali, Colombia

**Keywords:** bio-inspired navigation, robot guidance, obstacle avoidance, decision-making, motor control, ring attractor networks, social navigation

In the published article, there was an error in the article title. Instead of “*Ring attractor bio-inspired neural network for robot social navigation*”, it should be “*Ring attractor bio-inspired neural network for social robot navigation*”.

In the published article, there was an error in the author list, and authors Juan D. Hernández and Victor Romero-Cano were erroneously excluded. The corrected author list appears below.

Jesús D. Rivero-Ortega ^1*^, Juan S. Mosquera-Maturana ^1^, Josh Pardo-Cabrera ^1^, Julián Hurtado-López ^2*^, Juan D. Hernández ^3^, Victor Romero-Cano ^4,5^ and David F. Ramírez-Moreno ^6^

^3^School of Computer Science and Informatics, Cardiff University, Cardiff, United Kingdom

^4^Robotics and Autonomous Systems Laboratory, Faculty of Engineering, Universidad Autonoma de Occidente, Cali, Colombia

^5^Rimac Technology, Zagreb, Croatia

In the published article **Okal and Arras (2014)** was not cited in the article. The citation has now been inserted in **Materials and methods**, *Software*, *ROS Package*, Paragraph 1 and should read:

“To enable our neural network to control a robot interacting with a virtual environment, we adapted the IntegrationEngine class as a ROS node. The ROS version was Noetic on Ubuntu 20.04.5 LTS. The virtual simulations were performed on Gazebo 11. The system diagram is shown in Figure 2, where the different nodes and subsystems are depicted. The ROS_BINNF node performs the integration of the dynamical system that represents the neural network defined in Neuron.py. LiDAR information passes through the pc_regions_density node where it is clustered along 24 directions around the robot, resulting in the mean distance to obstacles in a certain direction. Then this is converted from an absolute to a relative coordinate system to then feed the obstacles-related information in the ROS_BINNF node. The PedSim ROS package (Okal and Arras, 2014) simulates the social agents in the simulation and sends this data for Gazebo to visualize them. As the follower agent can be simulated with or without PedSim ROS, he/she is not connected in the diagram. The target position is set in the target_setter node. In each integration step, the ROS_BINNF node sends information about the obstacles, target, and current state of the robot, along with the last step neural network's states to the Neuron class.”

In the published article **Silva et al. (2022, 2023)** was not cited in the article. The citation has now been inserted in **Materials and methods**, ***Framework structure***, Paragraph 1 and should read:

“The navigation system is divided into perception, planning, and control stages (as proposed in our previous work Silva et al., 2022, 2023). In the perception stage, data from the environment is obtained and processed. In the planning stage, the neural network is supplied with information about the environment and the goal position and generates control commands for the robot as output from the neural network. In the control stage, the signals coming from the network are decoded to determine the velocities for the differential control of the robot.”

In the published article **Hernández et al. (2016, 2019)** was not cited in the article. The citation has now been inserted in **Results**, ***Virtual robot simulation***, Paragraph 3 and should read:

“In order to obtain a socially acceptable behavior, paths were planned using Dubins curves that allowed straight-forward moves and right or left turns (as proposed in our previous work Hernández et al., 2016, 2019). To execute the planned paths, the angular and linear velocity of the robot were set to 1 m/s.”

In the published article, there was an error in the **Author Contributions**, and authors Juan David Hernández and Victor Romero-Cano were erroneously excluded. The corrected **Author Contributions** appears below.

“JR-O designed, implemented, and tested the proposed model. JM-M designed and implemented the social context simulation and metrics, and collected, compiled, and analyzed the simulation results. JH supervised the work related to social robot navigation, including the social context, its simulation, and benchmarking of the selected social metrics. VR-C supervised the work related to the use of social robot navigation for guiding social agents. JH-L and JP-C reviewed the state of the art. DR-M proposed the research topic and was in charge of guiding the workflow. DR-M and JH-L reviewed and read-proofed the manuscript. All authors contributed to the writing, editing, and formatting of the manuscript and approved the submitted version.”

In the published article, there was an error in the **acknowledgments**, some acknowledgments were erroneously excluded. The corrected **Acknowledgments** appears below.

“The authors are grateful to the Universidad Autónoma de Occidente and the Motor Neurocontrol Research Group. The authors would like to thank the Research group on remote and distributed control systems (GITCoD). The authors would like to thank Steven Silva, who guided the literature review in social robot navigation, as well as the required modifications in PedSim ROS. The authors also would like to thank the Editor and reviewers for their critical and constructive comments and suggestions.”

The authors apologize for this error and state that this does not change the scientific conclusions of the article in any way. The original article has been updated.
